# OSASUD: A dataset of stroke unit recordings for the detection of Obstructive Sleep Apnea Syndrome

**DOI:** 10.1038/s41597-022-01272-y

**Published:** 2022-04-19

**Authors:** Andrea Bernardini, Andrea Brunello, Gian Luigi Gigli, Angelo Montanari, Nicola Saccomanno

**Affiliations:** 1 Clinical Neurology Unit, Udine University Hospital, 33100 Udine, Italy; 2 Department of Mathematics, Computer Science, and Physics, University of Udine, 33100 Udine, Italy

**Keywords:** Sleep disorders, Stroke, Computer science

## Abstract

Polysomnography (PSG) is a fundamental diagnostical method for the detection of Obstructive Sleep Apnea Syndrome (OSAS). Historically, trained physicians have been manually identifying OSAS episodes in individuals based on PSG recordings. Such a task is highly important for stroke patients, since in such cases OSAS is linked to higher mortality and worse neurological deficits. Unfortunately, the number of strokes per day vastly outnumbers the availability of polysomnographs and dedicated healthcare professionals. The data in this work pertains to 30 patients that were admitted to the stroke unit of the Udine University Hospital, Italy. Unlike previous studies, exclusion criteria are minimal. As a result, data are strongly affected by noise, and individuals may suffer from several comorbidities. Each patient instance is composed of overnight vital signs data deriving from multi-channel ECG, photoplethysmography and polysomnography, and related domain expert’s OSAS annotations. The dataset aims to support the development of automated methods for the detection of OSAS events based on just routinely monitored vital signs, and capable of working in a real-world scenario.

## Abstract (summary)

**Table:** 

Measurement(s)	Nasal airflow • Snoring • Oxygen Saturation Measurement • Body Position • Thoracic movement • Abdominal movement • 3 Lead ECG Extracted From 12 Lead Continuous ECG Recording (http://ncicb.nci.nih.gov/xml/owl/EVS/Thesaurus.owl#C123445) • Heart rate • premature ventricular contractions AE • Respiratory Rate • Perfusion index • obstructive sleep apnea • photoplethysmography
Technology Type(s)	Pressure transducer connected to a nasal cannula • photoplethysmography • Accelerometer • Plethysmography • Ag-AgC1 electrodes, 12-lead (I, II, III, aVR, aVL, aVF, V1, V2, V3, V4, V5, V6) • Lead II ECG Ag-AgC1 electrodes • Physician’s annotation
Sample Characteristic - Organism	Homo sapiens
Sample Characteristic - Environment	hospital
Sample Characteristic - Location	Province of Udine

## Background & Summary

Obstructive Sleep Apnea Syndrome (OSAS) is one of the most common sleep-related breathing disorders^1^. It is caused by an increased upper airway resistance during sleep, that leads to periods of partial or complete interruption of airflow, bringing to reductions in blood oxygen content; these events are typically interrupted by arousals from sleep, with the result that OSAS commonly manifests itself with excessive daytime sleepiness due to sleep fragmentation; however, its most relevant health-related burden is represented by a higher risk of cardio- and cerebrovascular accidents such as myocardial infarction and ischemic stroke^2^.

OSAS is usually diagnosed by means of polysomnography (PSG)^3^, which requires overnight recording of at least the following parameters: airflow, blood oxygen saturation, thoracic and abdominal movements. In addition, other parameters are typically considered, such as: snoring, electrocardiography, electroencephalography, electrooculography, and surface electromyography of the mylohyoid and tibialis anterior muscles^4,5^. Such recordings are then manually tagged by a trained physician against the presence of apneic events^5^. As a result, performing a PSG is a labour-, time-, and money-consuming process.

The detection and treatment of OSAS are particularly important in stroke patients^6^. Stroke is defined as an episode of neurologic dysfunction due to infarction or focal collection of blood within the central nervous system^7^, and represents the second cause of death and the third cause of disability worldwide^8^. The optimal inpatient setting for acute stroke patients is represented by specialized semi-intensive care wards, named stroke units^9^. In a stroke unit, all patients undergo continuous monitoring of many vital parameters such as noninvasive blood pressure, multi-lead electrocardiography, photoplethysmography-derived blood oxygen saturation, and thoracic impedance-derived respiratory rate. OSAS is highly prevalent in stroke patients, with up to 91.2% of individuals being affected and 44.6% experiencing a severe condition^10^. The latter cases are prone to higher mortality, worse neurological deficits, worse functional outcome after rehabilitation, and a higher likelihood of uncontrolled hypertension^11,12^. Identifying and treating them is thus of fundamental importance.

Unfortunately, performing a PSG in an electrically hostile environment, such as a stroke unit, on neurologically impaired patients is a difficult task^13,14^, with the result that signals are often affected by noise; in addition, the number of strokes per day vastly outnumbers the availability of polysomnographs and dedicated healthcare professionals. Therefore, a simple and automated recognition system to identify OSAS cases among acute stroke patients is highly desirable. The continuous multiparametric recording of vital signs that is routinely performed in stroke units represents a relevant data source for a comprehensive assessment of a patient’s health status. However, such data represents an insufficient amount of information for traditional, manual sleep scoring^15^.

The dataset presented in this work (named OSASUD, Obstructive Sleep Apnea Stroke Unit Dataset) is aimed at supporting the development of automated methods for the identification of OSAS episodes based on simplified monitoring system data. It is composed of overnight recordings of 30 patients that were admitted to the stroke unit of the Udine University Hospital, Italy. For each patient, recordings of multi-channel ECG and photoplethysmography (PPG) are reported, together with derived data including heart rate, oxygen saturation, pulsatility index, respiratory rate, and premature ventricular contractions.

In the literature, several OSAS datasets have already been published with a similar goal, the most important ones being Physionet’s Apnea-ECG Database (35 training +35 test patients)^16^, SVUH/UCD St. Vincent’s University Hospital/University College Dublin Sleep Apnea Database (25 patients)^17^, HuGCDN2014 Database (77 patients)^18^, and MIT-BIH Polysomnographic Database (18 patients)^19^. Nevertheless, they are all far from representing a real-world situation: their data are recorded in ideal conditions and on highly selected patients, with stringent exclusion criteria concerning the presence of cardiac, respiratory, and other comorbidities. As a result, models developed according to them are hardly generalizable to real-life scenarios, where they would be of actual use.

Another source of sleep-related data, although not focused at apnea detection tasks, is the National Sleep Research Resource, which provides a repository^20^ of sleep study datasets, including the Sleep Heart Health Study (5804 subjects) and the MrOS Sleep Study (2911 subjects).

Our setting is quite different. The patients we consider show a considerably complex clinical situation, and the presence of comorbidities is the rule rather than the exception. In addition, recordings are affected by noise and missing data, as is typical in real-world monitoring systems. For these reasons, we believe that publicly sharing our dataset would represent a valid support for further advancing the research into OSAS detection.

## Methods

**Fig. 1:**
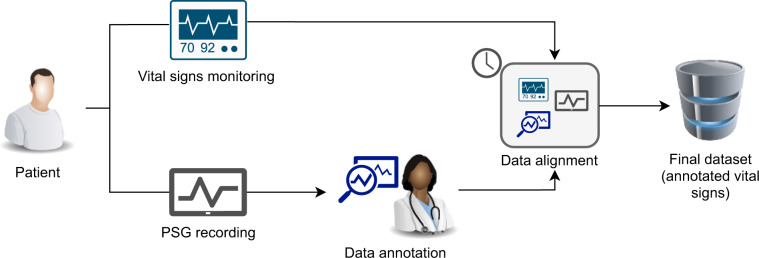
Workflow of the study.

Figure 1 depicts the overall workflow of the study. Each patient underwent simultaneous overnight PSG and vital signs (ECG and PPG) recording. The collected PSG data was then annotated by a trained sleep physician against the presence of apnea and hypopnea events, at one second granularity. The PSG data and annotations were then temporally aligned with and matched against the recorded vital signs. The final dataset was assembled considering the physician’s annotations and a relevant subset of the collected data. In the following, the different phases of the workflow are thoroughly described.

### Participants

The study consists of 30 patients who were admitted to the stroke unit of the Clinical Neurology Unit of the Udine University Hospital for a suspected cerebrovascular event (ischemic stroke, transient ischemic attack, or hemorrhagic stroke) from August 2019 to July 2020. Exclusion criteria were the following: age <18 years, insufficient compliance to standard monitoring and/or PSG, aphasia of sufficient severity to limit comprehension of the study protocol and/or expression of informed consent, high risk of alcohol/drug withdrawal syndrome. Diabetes mellitus, atrial fibrillation, cardiac disease, obesity, and other medical conditions not listed above were not considered as exclusion criteria. Table 1 reports detailed information regarding each patient. As can be seen, the data is quite heterogeneous considering age, gender, AHI, and quality of the recordings.

**Table 1:** Description of the patients considered in the study.

Patient	Age	Gender	BMI	Recording (hours)	# apneas	# hypopneas	(hypo)apnea duration (sec)	AHI
1	48	Male	30.8	7.0	142	136	22 ± 8	40
2	77	Male	35.3	11.9	29	93	20 ± 8	10
3	61	Male	33.2	7.1	242	208	25 ± 10	63
4	66	Male	26.9	9.0	52	42	34 ± 15	10
5	33	Male	35.9	9.1	42	280	23 ± 12	35
6	68	Male	45.0	4.1	87	150	20 ± 4	58
7	71	Male	30.8	9.2	72	203	16 ± 3	30
8	76	Female	25.0	9.0	3	10	17 ± 3	1
9	78	Female	29.4	9.5	24	53	18 ± 5	8
10	69	Male	27.8	11.4	265	207	21 ± 6	41
11	70	Male	26.7	8.4	10	28	21 ± 6	4
12	65	Male	25.0	8.4	24	9	20 ± 7	4
13	70	Male	33.1	9.5	22	220	19 ± 5	26
14	85	Female	25.1	10.3	9	78	31 ± 10	9
15	63	Male	35.3	9.2	235	162	29 ± 8	43
16	70	Male	31.9	9.0	269	62	34 ± 14	37
17	75	Female	24.8	8.1	96	129	22 ± 8	28
18	71	Female	16.8	10.0	38	6	15 ± 2	4
19	68	Female	28.2	9.5	10	82	21 ± 6	10
20	77	Male	24.6	9.8	335	132	26 ± 10	48
21	74	Male	26.1	9.8	216	61	22 ± 7	28
22	63	Male	29.1	8.0	7	6	26 ± 8	2
23	87	Female	20.4	9.5	1	2	19 ± 4	0
24	73	Male	32.0	9.8	28	181	26 ± 14	21
25	81	Female	32.0	10.6	41	428	19 ± 5	44
26	76	Female	22.3	9.0	425	115	23 ± 10	60
27	71	Female	27.7	7.6	50	21	19 ± 5	9
28	69	Female	27.3	7.4	8	89	29 ± 8	13
29	44	Male	26.6	7.8	2	32	17 ± 4	4
30	70	Male	40.1	8.2	263	336	19 ± 8	73

### Ethics declaration

All participants gave written informed consent prior to their participation to the study. The regional Ethics Committee (Comitato Etico Unico Regionale) of Friuli-Venezia Giulia, Italy approved the anonymous publication of data recordings.

### Data collection

Each patient underwent simultaneous overnight PSG and vital signs recording. Recordings were performed during the first days after clinical onset (average 1.31.1 days, range 0–5), while patients were still monitored in the Stroke Unit. Table 2 summarizes all collected signals.

**Table 2:** Summary of the signals collected for the study.

Source	Signal	Sampling rate (Hz)	Sensor type and placement	Preprocessing
Embletta	Nasal airflow	20	Pressure transducer connected to a nasal cannula	high-pass 0.1 Hz and low-pass 15 Hz filters
	Snoring	10	Derived from nasal airflow waveform data	none
	PPG	75	Red-infrared light-emitting diode and sensor positioned on the opposite sides of a finger	none
	Oxygen saturation	3	Derived from PPG data	none
	Body position	10	Three-axis accelerometer	none
	Thoracic movement	10	Respiratory inductance plethysmography band, positioned midway between the manubrium of the sternum and the xyphoid process	high-pass 0.1 Hz and low-pass 15 Hz filters
	Abdominal movement	10	Respiratory inductance plethysmography band, positioned midway the xyphoid process and the umbilicus	high-pass 0.1 Hz and low-pass 15 Hz filters
	ECG	500	Ag-AgC1 electrodes on the acromial head of each clavicle	high-pass 0.3 Hz and low-pass 70 Hz filters
	Heart rate	3	Derived from ECG data	none
Mindray	ECG	80	Ag-AgC1 electrodes, 12-lead (I, II, III, aVR, aVL, aVF, V1, V2, V3, V4, V5, V6)	high-pass 0.5 Hz and low-pass 40 Hz filters; 60 Hz notch filter
	Heart rate	1	Derived from ECG data	none
	Premature ventricular contractions	1	Derived from ECG data	none
	Thoracic impedance	80	Measured with lead II ECG Ag-AgC1 electrodes	high-pass 0.2 Hz and low-pass 2 Hz filters; 60 Hz notch filter
	Respiratory rate	1	Derived from thoracic impedance data	none
	PPG	80	Red-infrared light-emitting diode and sensor positioned on the opposite sides of a finger	none
	Pulse rate	1	Derived from PPG data	none
	Oxygen saturation	1	Derived from PPG data	none
	Perfusion index	1	Derived from PPG data	none
	Blood pressure	1/3600	Oscillometric arm cuff	none

A level 3 PSG without video recording was performed using an Embletta MPR polysomnograph (Natus Medical Inc., Pleasanton, CA, USA), keeping track of the following channels: nasal airflow, blood oxygen saturation, snoring, body position, thoracic and abdominal movements, and ECG. Nasal airflow was derived from a dedicated pressure transducer connected to a nasal cannula; sampling rate was 20 Hz, whereas high-pass and low-pass filters were set at 0.1 and 15 Hz respectively. Blood oxygen saturation was measured by means of transmission PPG with red-infrared light-emitting diode and sensor positioned on the opposite sides of a finger. The sampling rate for the PPG curve was 75 Hz; arterial oxygen saturation and heart rate were measured from the PPG signal with a 3 Hz sampling rate. Snoring intensity was estimated by means of nasal airflow waveform analysis, with a 10 Hz sampling rate. Body position was recorded with an internal three-axis accelerometer with a 10 Hz sampling rate; position data were also used to detect major body movements. Thoracic and abdominal movements were recorded by means of two independent respiratory inductance plethysmography single-use sensor bands; the thoracic band was positioned midway between the manubrium of the sternum and the xyphoid process, whereas the abdominal band was placed midway between the xyphoid process and the umbilicus. The sampling rate was 10 Hz for both channels, with high-pass and low-pass filters set at 0.1 and 15 Hz respectively. ECG was recorded with a single-use Ag-AgCl electrode on the acromial head of each clavicle, akin to a lead I with proximal electrode positioning^21^. The ECG signal was recorded with a 500 Hz sampling rate and high-pass and low-pass filters set at 0.3 and 70 Hz respectively. Files were analyzed with Embla RemLogic software, version 3.4.1.2371 (Natus Medical Inc., Pleasanton, CA, USA). Recordings were exported as EDF files^22^ with no gain adjustment or additional filtering; annotations were exported as separate TXT files with timestamps for each event.

Vital signs were collected by means of a Mindray iMec15 monitor connected to a Mindray Benevision CMS II central monitoring system (Mindray Bio-Medical Electronics Co., Ltd., Shenzhen). The following parameters were recorded: 12-lead ECG waveform (standard leads: I, II, III, aVR, aVL, aVF, V1, V2, V3, V4, V5, V6^21^) with single-use Ag-AgCl electrodes, ECG-derived heart rate, ECG-derived premature ventricular contraction (PVC) rate, thoracic impedance waveform measured from lead II electrodes, thoracic impedance-derived respiratory rate, PPG waveform recorded with red-infrared light-emitting diode and sensor positioned on the opposite sides of a finger, PPG-derived pulse rate, PPG-derived blood oxygen saturation, PPG-derived perfusion index, oscillometric arm cuff blood pressure (systolic, diastolic, and mean). Sampling frequencies were 80 Hz for ECG, PPG, and thoracic impedance waveforms, 1 Hz for heart rate, PVC rate, pulse rate, blood oxygen saturation, perfusion index, and respiratory rate, and 1/hour for blood pressure. Recording bandwidths (−3 dB) were 0.5–40 Hz for the ECG channels and 0.2–2 Hz for thoracic impedance, both with a 60 Hz notch filter. All data were exported from the central monitoring system storage disk as comma-separated value (CSV) files.

### Data annotation

**Fig. 2:**
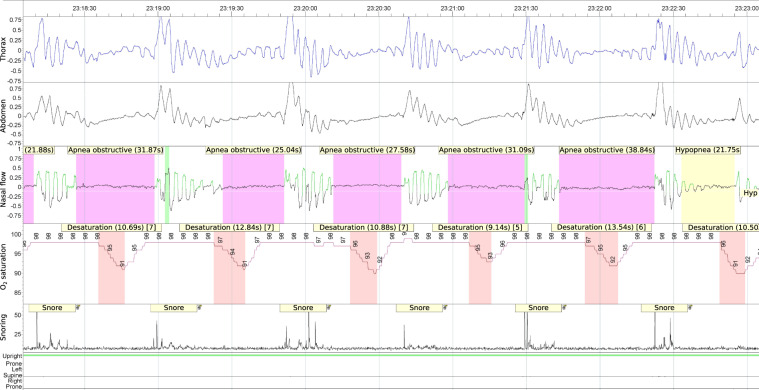
Polysomnographic recording tagged with different apnea events by means of Embla RemLogic software.

All PSG data were reviewed with Embla RemLogic software, version 3.4.1.2371 (Natus Medical Inc., Pleasanton, CA, USA), that allows for signal processing, inspection and annotation. The dataset was annotated by a trained sleep medicine physician in accordance with the American Academy of Sleep Medicine sleep scoring rules^15^, and tagged against the presence of central/obstructive/mixed apnea and hypopnea events (which we refer to as anomalies), each identified by its specific time interval. Figure 2 shows a partial recording with its annotations, opened in Embla RemLogic.

### Data transformation

**Fig. 3:**
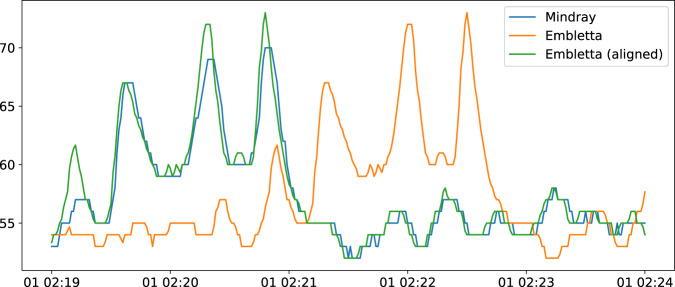
Embletta (original and aligned) and Mindray heart rate signals (5-minute interval).

Since patients’ data were simultaneously recorded by means of two different devices (the Embletta polysomnograph and the Mindray monitoring system), they needed to be temporally aligned. This is quite natural, as different devices may have slightly different clocks which they use to timestamp the data (i.e., the same timestamp, on different devices, might refer to slightly different real-world time instants). Given a patient, to determine the time shift between its two sets of recordings, we proceeded as follows. We considered Embletta’s and Mindray’s oxygen saturation and heart rate recordings, downsampling Embletta’s data from 3 Hz to 1 Hz by means of averaging. We calculated the correlation between the two heart rate time series at different time offsets. We then repeated the same process for the two oxygen saturation time series. As a result, we obtained two different shift estimates. Starting from the smallest estimate, we ultimately fine-tuned the shift value by hand looking at different parts of the signals, obtaining the final alignment. Figure 3 shows the situation for the heart rate signal of one of the considered patients, before and after the alignment process.

Thanks to the alignment process, we were then able to correctly associate the PSG data and related apnea annotations performed by the physician to the Mindray data. Next, values of oxygen saturation below 50 or above 100 were considered to be artifacts, and assigned to null. The same approach was taken for values of heart rate below 20 or above 200, and respiratory rate below 5 or above 40. Whenever oxygen saturation was set to null, we also set perfusion index to null. To the value of premature ventricular contractions we set an upper bound equal to the corresponding heart rate. As for PSG data related to airflow, snoring, body position, thoracic and abdominal movements, we standardized each signal individually for each patient. This allows us to improve their comparability, since different calibrations are expected to be used for different recording sessions. Finally, data were de-identified in order to preserve the privacy of the participants. Observe that no signal filtering was applied in this phase.

## Data Records

The dataset OSASUD consists of a Pandas^23^ DataFrame with 18 columns and 961357 rows, saved in Pickle format (file *dataset_OSAS.pickle*^24^). Table 3 provides an overview of the columns. Observe that we consider only a subset of the originally recorded data. The reason is two-fold: (*i*) some signals are redundant, for instance, the PPG and ECG waveforms. In such cases, given the aim of our dataset, we favour Mindray data; and, (*ii*) some signals are only used for auxiliary tasks, for example, this is the case of thoracic impedance, from which respiratory rate is derived.

**Table 3:** Description of the columns in the dataset.

Column Name	Format	Description
patient	String	Participant ID
timestamp_datetime	Datetime YYYY-MM-DD HH:MM:SS	Date and time (at one second granularity) of the recorded data
HR(bpm)	Float64	ECG-derived heart rate
SpO2(%)	Float64	PPG-derived oxygen saturation, in %
PI(%)	Float64	PPG-derived perfusion index
RR(rpm)	Float64	ECG-derived respiratory rate (per minute)
PVCs(/min)	Float64	ECG-derived premature ventricular contractions (per minute)
event	String	A string among: ‘NONE’, ‘HYPOPNEA’, ‘APNEA-CENTRAL’, ‘APNEA-OBSTRUCTIVE’, ‘APNEA-MIXED’
anomaly	Boolean	True = anomaly present (either apnea or hypopnea), False = no anomaly present (i.e., event = ‘NONE’)
signal_pleth	Array of Float64	80 samples of waveform PPG signal
signal_ecg_i	Array of Float64	80 samples of waveform ECG signal, lead I
signal_ecg_ii	Array of Float64	80 samples of waveform ECG signal, lead II
signal_ecg_iii	Array of Float64	80 samples of waveform ECG signal, lead III
PSG_Abdomen	Array of Float64	10 samples of abdominal movement signal
PSG_Flow	Array of Float64	20 samples of nasal airflow signal
PSG_Position	Array of Float64	10 samples of body position signal
PSG_Snore	Array of Float64	10 samples of snoring signal
PSG_Thorax	Array of Float64	10 samples of thoracic movement signal

As a result, each row is characterized by an anonymous identifier of the patient and a timestamp that keeps track of the time instant at which the data was recorded, at one second granularity. As for the other columns, they report:

- the ECG-derived heart rate, respiratory rate, and premature ventricular contractions per minute;
- the PPG-derived oxygen saturation (in %) and perfusion index (in %);
- the physician’s annotation, that distinguishes between regular breathing behaviour (string ‘NONE’), hypopnea (string ‘HYPOPNEA’), and different kinds of apnea (strings ‘APNEA-CENTRAL’, ‘APNEA-OBSTRUCTIVE’, ‘APNEA-MIXED’);
- a boolean attribute that coarsely distinguishes between regular and anomalous breathing behaviour (it equals *True* if and only if the annotation is not ‘NONE’);
- a waveform PPG signal, composed of 80 values (given the 80 Hz sampling rate);
- three ECG waveform signals referring to the leads I, II, and III, each composed of 80 values (again, given the 80 Hz sampling rate);
- the signals recorded by PSG, referred to nasal airflow (20 values, given the 20 Hz sampling rate), snoring (10 values), body position (10 values), thoracic and abdominal movements (10 values each).

Note that, given a patient, its data are contiguous from the start to the end of her/his overnight recording. In the event in which values were missing for a time instant, they were replaced by null (constant *numpy.NaN*^25^) in order to maintain timestamp contiguity.

## Technical Validation

For each patient, we determined the amount of null values in PPG and ECG waveforms, and their derived attributes. Results are presented in Table 4. Annotations are always present (with ‘NONE’ as the default value), as well as PSG-recorded signals.

**Table 4:** Null values in PPG and ECG waveforms, and their derived attributes.

Patient	HR(bpm)	SpO2(%)	PI(%)	RR(rpm)	PVCs(/min)	signal_pleth	signal_ecg_i	signal_ecg_ii	signal_ecg_iii
1	0.0	8.3	8.5	0.0	0.0	8.2	0.0	0.0	0.0
2	0.0	6.1	6.1	0.0	0.0	6.1	0.3	0.0	53.8
3	0.0	0.2	0.4	2.7	0.0	0.0	0.0	0.0	0.0
4	0.3	0.2	0.3	0.7	0.3	0.2	1.4	0.2	1.4
5	1.9	3.6	3.6	1.6	1.9	3.4	1.5	1.4	1.5
6	0.9	1.6	1.9	0.1	0.9	0.0	0.0	0.0	0.0
7	0.0	11.1	11.2	1.2	0.0	10.9	0.0	0.0	0.0
8	0.0	12.1	12.1	0.0	0.0	11.8	0.0	0.0	0.0
9	0.3	13.4	13.5	0.4	0.3	13.3	0.3	0.3	0.3
10	5.8	17.8	17.9	5.9	5.8	17.5	5.8	5.8	5.8
11	0.0	22.1	22.2	0.0	0.0	21.5	0.0	0.0	0.0
12	0.0	0.0	0.0	1.5	0.0	0.0	0.0	0.0	0.0
13	0.0	1.9	2.0	0.2	0.0	1.8	0.0	0.0	0.0
14	0.0	2.6	2.7	0.1	0.0	2.5	0.0	0.0	0.0
15	0.2	19.1	19.1	0.6	0.2	19.0	0.0	0.0	0.0
16	0.0	11.9	12.1	4.4	0.0	11.1	0.0	11.8	11.8
17	0.0	65.7	65.8	0.2	0.0	65.6	0.0	0.0	0.0
18	0.0	37.3	37.4	0.0	0.0	37.0	0.0	0.0	0.0
19	0.0	16.8	16.9	0.0	0.0	16.7	0.0	0.0	0.0
20	0.0	3.9	3.9	11.0	0.0	3.8	0.0	0.0	0.0
21	0.1	0.1	0.3	0.3	0.1	0.0	0.0	0.0	0.0
22	0.0	22.4	22.5	12.4	0.0	21.7	0.0	0.0	0.0
23	0.0	0.8	0.8	11.9	0.0	0.8	0.0	0.0	0.0
24	0.0	12.2	12.3	0.0	0.0	11.9	0.0	0.0	0.0
25	0.0	18.4	18.4	0.2	0.0	18.2	0.0	0.0	0.0
26	0.0	12.7	12.7	0.0	0.0	12.6	0.0	0.0	0.0
27	0.0	15.1	15.2	0.2	0.0	14.9	0.0	0.0	0.0
28	0.0	72.7	72.7	0.0	0.0	72.6	0.0	0.0	0.0
29	0.0	9.9	10.0	0.0	0.0	9.8	0.0	0.0	0.0
30	0.0	0.5	0.6	0.0	0.0	0.0	0.0	0.0	0.0

As for the non-null values, the acquired PPG and ECG waveforms, their derived data, and the PSG-recorded signals were carefully inspected by a trained physician jointly with the PSG-based apnea events annotation phase. From the inspection, it resulted that several recordings were affected by artifacts, either caused by the presence of comorbidities (e.g., atrial fibrillation) or sudden movements performed by the patients. Such artifacts are unavoidable and common during recordings in a clinical setting, especially in an electrically hostile environment such as an intensive care unit. Given our dataset’s purpose of modelling a real-world scenario, we chose to keep all the data, without removing noisy or null segments.

**Fig. 4:**
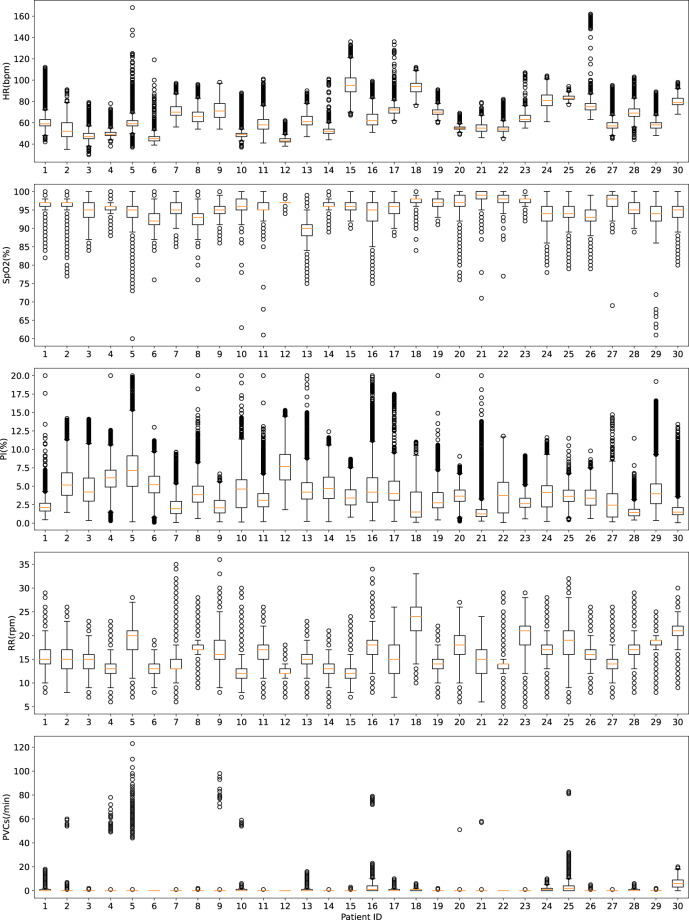
Value distribution for the ECG- and PPG- derived attributes in the dataset (excluding null values).

Figure 4 presents the value distribution for each patient and ECG- and PPG- derived attributes. Null values have been ignored. Each box extends from the first to the third quartile values of the data, with a line at the median. Whiskers extend to the smallest and largest observations which are not outliers (considering 1.5 times the interquartile range).

A final validation of the dataset comes from the successful development of a deep learning model for OSAS event prediction based on the considered data, recently presented in the literature^26^.

## Usage Notes

We have successfully read the dataset loading it by means of Python’s Pickle and Pandas packages. As each row of the Pandas DataFrame contains one second-worth of data pertaining to a given patient, in order to produce a machine learning training dataset to support the detection of OSAS based on data recorded by ECG and PPG, we suggest the following processing.

For each patient, concatenate all of its rows column-wise, so to obtain the full time series pertaining to each of the data columns. Since no filtering was performed on the waveform signals, we also suggest to apply a Butterworth bandpass filter of order 2, with 5 Hz highpass frequency and 35 Hz lowpass frequency on the ECG and PPG waveform time series. At this point, divide each time series based on a *s*-second windowing approach, possibly with a certain degree of overlap between the windows. As a result, each instance is characterized by five *s*-second windows related to the ECG and PPG derived data, three (*s*·80)-second windows concerning the waveform data, and two *s*-second windows containing the apnea event labels, respectively coded as a string or a boolean value. Finally, possibly remove those windows in which all predictors exhibit more than a specific degree (e.g., 50%) of null values. Note that, since raw waveforms have been included in the dataset, derived data other than those already provided can be easily calculated. This is the case for instance of features pertaining to the QRS complex, or to the pulse transit time.

We encourage using the dataset for the development of automated (either statistical or machine learning-based) solutions, for instance in the following scenarios:

- a model can be trained to predict the presence or absence of breathing anomalies based on the attribute *anomaly*, or to derive a more detailed classification by means of the attribute *event*. Note that, given the nature of the dataset, contrary to most previously published data sources, predictions at one-second granularity are possible, i.e., a model can be trained to determine the exact start and end time of each OSAS event;
- observe that sleep-disordered breathing occurring in the acute setting of cerebrovascular disease may present different features from sleep-disordered breathing in the general population. This database offers researchers a tool to train or test models for the identification of respiratory events in this specific subset of patients. In addition, given the real-world connotation of our dataset, it could be used to develop and embed models in current monitoring systems, with the aim of identifying sleep-disordered breathing in acute stroke patients, without resorting to mass PSG screening;
- an unsupervised model trained to detect unexpected signal variations emerging from the background variability may be considered, with the idea that such variations may act as a biomarker of clinical instability;
- thanks to the inclusion of detailed polysomnographic data, the dataset may also support studies aimed at uncovering qualitative and quantitative relationships between PSG-, PPG-, and ECG-derived information.

It should be noted that our work still presents some limitations: first of all, the sample size is relatively small. Second, all recordings have been performed at a single center. Moreover, all included patients share some homogeneous characteristics regarding ethnicity, region of origin and reason for admission. Additionally, we performed PSG with a class III device, which does not include EEG channels. Therefore, we could not obtain a proper sleep staging nor identify arousals: periods of wake after sleep onset and hypopneas associated with arousals but without significant desaturation may have been missed. Finally, all recordings have been performed once within the first days after disease onset, with no follow-up recordings or later acquisitions for comparison.

## Data Availability

To allow for an easier usage of our data, a Python Jupyter Notebook is also included with the dataset (file *preprocess_dataset.ipynb*^24^). The notebook has been tested with the following packages versions: pandas = 1.3.3, numpy = 1.20.3, pickle = 4.0. The code performs a series of data pre-processing operations, that include: • loading the Pickle file that encodes the dataset as a Pandas DataFrame; • printing some validation results, including the values presented in Table 1; • generating a sample machine learning-ready dataset, in the form of a set of Numpy arrays. The code provided significantly contributes to relieve the burden of data pre-processing, which typically absorbs a major part of time in the development and testing of machine learning solutions.

## References

[1] Senaratna CV (2017). Prevalence of obstructive sleep apnea in the general population: A systematic review. Sleep Medicine Reviews.

[2] Sánchez-de-la Torre M, Campos-Rodriguez F, Barbé F (2013). Obstructive Sleep Apnoea and Cardiovascular Disease. Lancet Respiratory Medicine.

[3] American Academy of Sleep Medicine. *International Classification of Sleep Disorders*, 3 edn (American Academy of Sleep Medicine, 2014).

[4] Kapur VK (2017). Clinical Practice Guideline for Diagnostic Testing for Adult Obstructive Sleep Apnea: An American Academy of Sleep Medicine Clinical Practice Guideline. Journal of Clinical Sleep Medicine.

[5] Berry, R. *et al*. *The AASM Manual for the Scoring of Sleep and Associated Events: Rules, Terminology and Technical Specifications*, 2.6 edn (American Academy of Sleep Medicine, 2020).

[6] Brill AK (2018). CPAP as Treatment of Sleep Apnea After Stroke: A Meta-analysis of Randomized Trials. Neurology.

[7] Sacco RL (2013). An Updated Definition of Stroke for the 21st Century: A Statement for Healthcare Professionals from the American Heart Association/American Stroke Association. Stroke.

[8] Institute for Health Metrics and Evaluation. Global Burden of Disease 2017. https://vizhub.healthdata.org/gbd-compare/. Accessed: 2020-03-12.

[9] Powers WJ (2018). 2018 Guidelines for the Early Management of Patients With Acute Ischemic Stroke: A Guideline for Healthcare Professionals From the American Heart Association/American Stroke Association. Stroke.

[10] Huhtakangas JK, Huhtakangas J, Bloigu R, Saaresranta T (2017). Prevalence of Sleep Apnea at the Acute Phase of Ischemic Stroke with or without Thrombolysis. Sleep Medicine.

[11] Kumar R, Suri JC, Manocha R (2017). Study of Association of Severity of Sleep Disordered Breathing and Functional Outcome in Stroke Patients. Sleep Medicine.

[12] Xie W, Zheng F, Song X (2014). Obstructive Sleep Apnea and Serious Adverse Outcomes in Patients with Cardiovascular or Cerebrovascular Disease: A PRISMA-compliant Systematic Review and Meta-analysis. Medicine (Baltimore).

[13] Brown DL (2013). Sleep apnea treatment after stroke (SATS) trial: is it feasible?. Journal of Stroke and Cerebrovascular Diseases.

[14] Weinhouse GL, Kimchi E, Watson P, Devlin JW (2022). Sleep Assessment in Critically Ill Adults: Established Methods and Emerging Strategies. Critical Care Explorations.

[15] Berry RB (2012). Rules for scoring respiratory events in sleep: update of the 2007 AASM Manual for the Scoring of Sleep and Associated Events. Deliberations of the Sleep Apnea Definitions Task Force of the American Academy of Sleep Medicine. Journal of Clinical Sleep Medicine.

[16] Penzel, T., Moody, G. B., Mark, R. G., Goldberger, A. L. & Peter, J. H. The Apnea-ECG database. In *Computers in Cardiology*, **27**, 255–258, 10.13026/C23W2R (IEEE, 2000).

[17] St Heneghan C (2011). PhysioNet.

[18] Juliá-Serdá G, Navarro-Esteva J, Ravelo-García AG (2018). Mendeley Data.

[19] Ichimaru Y, Moody G (1999). Development of the polysomnographic database on CD-ROM. Psychiatry and Clinical Neurosciences.

[20] National Sleep Research Resource. NSRR sleep dataset repository. https://sleepdata.org/datasets/.

[21] Kligfield P (2007). Recommendations for the standardization and interpretation of the electrocardiogram: part I: The electrocardiogram and its technology: a scientific statement from the American Heart Association Electrocardiography and Arrhythmias Committee, Council on Clinical Cardiology; the American College of Cardiology Foundation; and the Heart Rhythm Society: endorsed by the International Society for Computerized Electrocardiology. Circulation.

[22] Kemp B, Värri A, Rosa AC, Nielsen KD, Gade J (1992). A simple format for exchange of digitized polygraphic recordings. Electroencephalography and Clinical Neurophysiology.

[23] Wes McKinney. Data structures for statistical computing in Python. In S. van der Walt & J. Millman (eds.) *Proceedings of the 9th Python in Science Conference*, 56–61 (2010).

[24] Bernardini A, Brunello A, Gigli GL, Montanari A, Saccomanno NA (2022). figshare.

[25] Harris CR (2020). Array programming with NumPy. Nature.

[26] Bernardini, A., Brunello, A., Gigli, G. L., Montanari, A. & Saccomanno, N. AIOSA: An approach to the automatic identification of obstructive sleep apnea events based on deep learning. *Artificial Intelligence in Medicine* 102133 (2021). 10.1016/j.artmed.2021.102133 34412849

